# Estimating the impact of expanded access to antiretroviral therapy on maternal, paternal and double orphans in sub-Saharan Africa, 2009-2020

**DOI:** 10.1186/1742-6405-8-13

**Published:** 2011-03-07

**Authors:** Aranka Anema, Christopher G Au-Yeung, Michel Joffres, Angela Kaida, Krisztina Vasarhelyi, Steve Kanters, Julio SG Montaner, Robert S Hogg

**Affiliations:** 1 British Columbia Centre for Excellence in HIV/AIDS, St. Paul's Hospital, Vancouver, British Columbia, Canada; 2 Faculty of Medicine, University of British Columbia, Vancouver, British Columbia, Canada; 3 Faculty of Health Sciences, Simon Fraser University, Burnaby, British Columbia, Canada; 4 Inter-disciplinary Research for Mathematical and Computational Sciences (IRMACS), Simon Fraser University, Burnaby, British Columbia, Canada

## Abstract

**Background:**

HIV/AIDS has orphaned 11.6 million children in sub-Saharan Africa. Expanded antiretroviral therapy (ART) use may reduce AIDS orphanhood by decreasing adult mortality and population-level HIV transmission.

**Methods:**

We modeled two scenarios to measure the impact of adult ART use on the incidence of orphanhood in 10 sub-Saharan African countries, from 2009 to 2020. Demographic model data inputs were obtained from cohort studies, UNAIDS, UN Population Division, WHO and the US Census Bureau.

**Results:**

Compared to current rates of ART uptake, universal ART access averted 4.37 million more AIDS orphans by year 2020, including 3.15 million maternal, 1.89 million paternal and 0.75 million double orphans. The number of AIDS orphans averted was highest in South Africa (901.71 thousand) and Nigeria (839.01 thousand), and lowest in Zimbabwe (86.96 thousand) and Côte d'Ivoire (109.12 thousand).

**Conclusion:**

Universal ART use may significantly reduce orphanhood in sub-Saharan Africa.

## Introduction

An estimated 11.6 million children (aged 0 to 17 years) in sub-Saharan Africa have lost one or both parents due to human immunodeficiency virus/acquired immune deficiency syndrome (HIV/AIDS) since the beginning of the epidemic [1]. Studies suggest that orphans in sub-Saharan Africa may have poor quality of life and health, including reduced access to basic material goods and retention in education [2], and elevated psychological distress and symptoms of depression [3,4]. Orphans may be at heightened risk of acquiring HIV due to engagement in early and unprotected sex, and in multiple sexual relationships [5,6]. HIV-infected orphans have shown to have delayed access to HIV treatment and care, reduced adherence to HIV treatment, and poor nutritional status [7-9].

Antiretroviral therapy (ART) has substantially reduced HIV-related morbidity and mortality worldwide [10]. A growing body of empirical evidence and mathematical modeling suggests that expanded ART use may also prevent population-level transmission of HIV [11-14]. In sub-Saharan Africa, 44% (2.925 million) of people clinically eligible for treatment were receiving it at the end of 2008 [15]. Several studies have evaluated the impact of the AIDS epidemic on orphanhood [15,16]. However, none to date have examined this in the context of efforts to expand ART access. We sought to determine to what extent the varying rates of ART uptake among adults would prevent the incidence of paternal, maternal and dual orphans in sub-Saharan Africa, from 2009 to 2020.

## Methods

We projected the impact of ART expansion to adults (15-49 years) on the incidence of paternal, maternal and dual orphans in 10 sub-Saharan African countries, from 2009 to 2020. We included 10 sub-Saharan African countries with the highest number of AIDS orphans living in 2007: Cote D'Ivoire, Ethiopia, Kenya, Malawi, Nigeria, South Africa, Uganda, United Republic of Tanzania, Zambia, and Zimbabwe [1].

In order to explore the impact of expanded ART use on orphanhood, we modeled two scenarios. Scenario 1 theoretically assumed that all (100%) HIV infected adults in the countries under study would receive ART immediately after year 2008, irrespective of CD4+ cell count or clinical stage. Scenario 2 assumed that the number of adults receiving ART remained constant following year 2008, reflecting country-specific rates of ART uptake and clinical eligibility of people living with HIV/AIDS in that year [15].

These scenarios were developed using DemProj and AIM programs within the Spectrum Policy Modeling System (Futures Group International) software package, Version 3.34. These programs are designed to produce information that is useful for policy formulation and program planning, and have been used by UNAIDS, UNICEF, USAID and the US Census Bureau to estimate orphanhood. Detailed descriptions of how Spectrum models the impact of HIV/AIDS on demographic parameters, including background mathematical methodology and parameter estimates, are described elsewhere [17-26]. We followed the prescribed steps for making HIV/AIDS and orphanhood projections, as outlined in the USAID Health Policy Initiative's recent guidelines [21,23]

Country-specific demographic and epidemiological model inputs are described in Table 1. All inputs and parameters used default values in the Spectrum program developed by the UNAIDS References Group on Estimates, Model and Projections [23]. Where possible, default values were exchanged with more recent empirical data, as described below.

**Table 1:** Country-specific projection model inputs

	**Number of single and dual AIDS orphans (0-17 yrs), 2007 **[44]	**HIV prevalence, adults 15-49 yrs, 2007 (%) **[44]	**Estimated annual increase in number of people receiving ART, 2008 **[15]	Reported Number HIV+ people receiving **ART, 2008 **[15]	**Number of HIV+ pregnant women receiving ART for PMTCT, 2008 **[15]	Estimated Number of HIV+ pregnant women who need ART, 2008 [15]
**South Africa**	1,400,000	18.1	192,840	700,500	149,118	200,000

**Uganda**	1,200,000	5.4	42,492	153,718	41,598	82,000

**Nigeria**	1,200,000	3.1	68,544	238,659	19,804	210,000

**Kenya**	1,195,000	7.8	65,880	242,881	59,601	110,000

**Zimbabwe**	1,000,000	15.3	50,112	147,804	18,756	53,000

**United Rep. of Tanzania**	970,000	6.2	18,768	154,468	70,944	85,000

**Ethiopia**	650,000	2.1	42 168	132,379	6,354	36,000

**Zambia**	600,000	15.2	74,436	225,634	41,286	70,000

**Malawi**	560,000	11.9	46,008	146,657	33,838	57,000

**Cote d'Ivoire**	420,000	3.9	13,608	51,833	9,296	22,000

### Non-HIV demographic inputs

As a first step to developing our AIDS orphanhood projection model, we conducted a demographic projection. This involved inputting non-HIV country-specific demographic estimates, such as population size, fertility and life expectancy, into the Spectrum Policy Modeling System's DemProj Program.

#### Population estimates

Country and age-specific population estimates for each year were obtained from the United Nations Population Division. In order to ensure consistency between population sizes from our demographic projections and country-specific census estimates, some of our demographic inputs were obtained from the US Census Bureau instead of the United Nations Population Division [27]. This process of matching current population estimates with projection outputs is described elsewhere [23,28,29].

#### Fertility estimates

We obtained country- and age-specific total fertility rates (TFR) from the US Census Bureau's World Population Profile [30]. The age distribution of fertility was estimated using the United Nations Sub-Saharan Africa model fertility table as outlined by Spectrum.

#### Mortality estimates

For non-HIV infected individuals, we inputted age-specific distributions of life expectancy at birth for non-AIDS-related mortality using the DemProj feature of Spectrum.

### HIV-specific inputs

#### HIV-specific fertility

A review and meta-analysis of 19 studies examining the population-level impact of HIV on fertility in sub-Saharan Africa reported that HIV-positive women not receiving ART have substantially lower TFR compared to HIV-negative women. This fertility differential resulted in a 0.37% decrease in population-attributable fertility for each percentage point of HIV prevalence within a country [31]. In order to incorporate this reduction in TFR in HIV-infected women into our projections, we used the default TFR reduction feature in AIM, which inputs age-specific ratios of fertility for HIV infected women compared to fertility in uninfected women.

#### HIV incidence

Country-specific HIV incidence inputs for adults (15-49 years) for years 1985 to 2008 were obtained using the UNAIDS-developed Estimation and Projection Package (EPP) software, and were converted into percentages before being inputted into the AIM program [32]. We assumed HIV was transmitted vertically and through heterosexual contact. We assumed individuals receiving ART were on triple combination therapy, or ART. In Scenario 1, we assumed that individuals receiving ART had suppressed HIV plasma viral load [14]. Based on empirical results from a study in Rakai, Uganda, we assumed that no cases of HIV transmission occurred among discordant contacts [33], and assumed HIV incidence was zero for every year subsequent to 2008. In Scenario 2, we assumed HIV incidence remained at the country-specific rate for 2008, reflecting current rates of ART uptake [15].

#### HIV disease progression and survival

We inputted varying disease progression data for Scenarios 1 and 2. In Scenario 1, we assumed that all HIV infected individuals were clinically eligible to receive ART from end 2008 onward [15]. In Scenario 2, we assumed that individuals were clinical eligible for ART if they had CD4 cell count under 350, and that time from HIV infection to ART eligibility was 3.2 years [23].

For individuals not receiving ART, we assumed that the median time from HIV infection to AIDS death, without treatment, was 10.5 years for men and 11.5 years for women [23]. These assumptions were based on findings from a large multi-country cohort study in low-resource settings [34]. For adults on ART, we assumed a survival rate of 0.86 for the first year on ART. This figure was derived from longitudinal cohort studies and systematic review of ART patients in low-incomes settings, and are recommended for use by the AIM projection model guidelines [23]. The survival rate of individuals receiving ART gradually increased over a 5-year period, and remained constant at 0.94 for the duration of the study period, based on a multi-country prospective cohort across low-income settings [35]. However, due to limitations in Spectrum, the survival rate for adults on ART was capped at 0.93 in subsequent years.

#### ART and PMTCT uptake

In Scenario 1, we assumed that all HIV-positive individuals were receiving ART as of year 2009. In Scenario 2, we inputted country-specific estimates for annual ART uptake, based on UNAIDS 2008 figures [15]. We assumed that antiretroviral (ARV) prophylaxis was unavailable to HIV-positive pregnant women in our countries of interest prior to the year 2004 and that it was entirely triple ARV prophylaxis. For Scenario 1, we assumed that all HIV-positive pregnant women received ARV prophylaxis for prevention of mother-to-child-transmission (PMTCT) from year 2009 onward. For Scenario 2, we inputted the percentage of HIV-positive pregnant women receiving PMTCT between the years 2004 and 2008 obtained from UNAIDS country-specific epidemiological fact sheets [15,36]. Other inputs under the Mother to Child Transmission section of AIM were unaltered.

### Outcomes variables

Our primary outcomes were the number of maternal, paternal and dual AIDS orphans in each country at year 2020 following varying scenarios of ART uptake. Maternal and paternal AIDS orphans were defined as children under the age of 17 who have lost either their mother or father to AIDS. Dual orphans are children who have lost both parents to AIDS [23].

### Projection and Calibration of Model

We ran each country's DemProj and AIM input data together from Spectrum to project the number of AIDS orphans incurred in each year. In order to calibrate our model, we ran DemProj and AIM programs for each country, using the above inputted data and parameters, from 1985 to end 2007. We verified the accuracy of our AIDS orphans projections by comparing our results for 2007 to the 2007 AIDS orphan estimate published in UNAIDS country-specific epidemiological fact sheets [36]. In order to identify the best fit for our model, as described in previous sections, we modified assumptions regarding population size and HIV survival rates using published ranges for census [23,27-29] and empirical cohort [23,34,35] data.

## Results

Table 2 presents the projected number of maternal, paternal, double and total AIDS orphans averted, per sub-Saharan African country, by varying levels of ART uptake at year 2020. Scenario 1, in which adults had universal ART access, averted a cumulative total of 4.37 million more AIDS orphans by year 2020 than Scenario 2, where ART access was expanded gradually. This included an estimated 3.15 million maternal orphans, 1.89 million paternal orphans and 748,320 double orphans.

**Table 2:** Projected number of maternal, paternal, and double AIDS orphans incurred and averted, per sub-Saharan African country, at year 2020

	South Africa	Uganda	Nigeria	Kenya	Zimbabwe	Tanzania	Ethiopia	Zambia	Malawi	Cote d'Ivoire
Orphans incurred with universal ART access										

Maternal	1,379,420	379,000	887,810	691,022	286,624	549,876	316,258	413,474	312,314	151,461

Paternal	1,452,297	592,386	1,165,760	913,492	410,784	735,112	421,703	535,465	432,220	241,890

Double	688,762	151,493	201,155	378,776	171,243	233,989	77,547	224,763	126,391	77,932

All	2,258,756	857,842	1,982,969	1,288,338	561,259	1,096,206	693,419	769,052	632,518	325,891

Orphans incurred by sustaining current ART access										

Maternal	2,258,756	567,307	1,413,087	1,175,760	362,142	883,904	497,135	641,775	491,560	224,070

Paternal	1,813,896	735,912	1,501,877	1,238,024	433,892	1,008,982	547,186	663,933	557,547	288,159

Double	940,552	192,112	282,339	503,190	188,051	318,425	98,724	290,848	174,617	91,513

All	3,160,461	1,163,017	2,821,983	2,005,720	648,220	1,641,721	994,221	1,075,967	894,946	435,012

Orphans averted with universal ART access										

Maternal	879,336	188,307	525,277	484,738	75,518	334,028	180,877	228,301	179,246	72,609

Paternal	361,599	143,526	336,117	324,532	23,108	273,870	125,483	128,468	125,327	46,269

Double	251,790	40,619	81,184	124,414	16,808	84,436	21,177	66,085	48,226	13,581

All	901,705	305,175	839,014	717,382	86,961	545,515	300,802	306,915	262,428	109,121

Countries with the largest number of AIDS orphans averted over the study period included South Africa (901,705), Nigeria (839,014), and Kenya (717,382). Countries with the least number of AIDS orphans averted were Zimbabwe (86,961), Malawi (262,428) and Côte d'Ivoire (109,121). The number of maternal orphans averted was higher than the number of paternal orphans averted in all countries: South Africa (879,336 *versus *361,599), Uganda (188,307 *versus* 143,526), Nigeria (525,277 *versus *336,117), Kenya (484, 738 *versus *324,532), Zimbabwe (75,518 *versus *23,108), Tanzania (334,028 *versus *273,870), Ethiopia (180,877 *versus *125,483), Zambia (228,301 *versus *128,468), Malawi (179,246 *versus *125,327), and Cote d'Ivoire (72,609 *versus *46,269)

**Figure 1:**
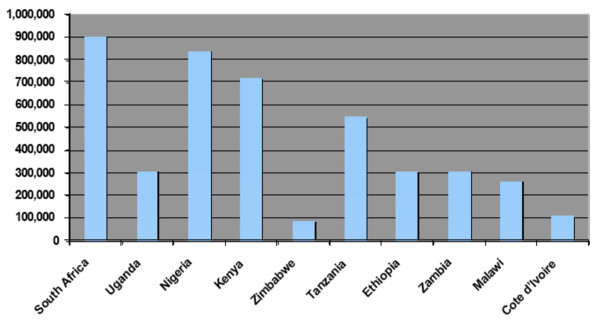
**Maternal, paternal, and double AIDS orphans averted due to universal antiretroviral uptake in ten Sub-Saharan African countries by year 2020**.

Figure 1 describes the number of maternal, paternal, and double AIDS orphans averted at year 2020, by country, due to universal ART access. It shows that the number of total AIDS orphans averted by increasing ART access would be highest in South Africa (901,705) and lowest in Zimbabwe (86,961).

**Figure 2:**
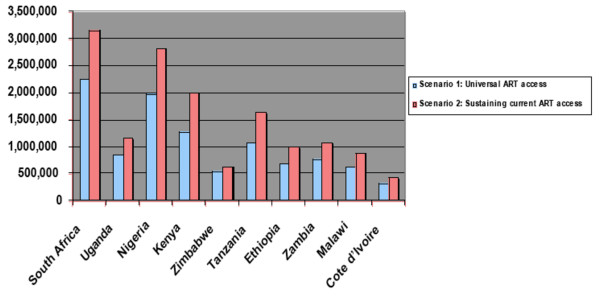
**Total number of AIDS orphans incurred in Scenario 1 (Universal ART uptake) and Scenario 2 (Sustaining current rate of ART access) in 10 Sub-Saharan African countries by year 2020**.

Figure 2 shows the number of orphans incurred in Scenario 1 and Scenario 2 for each of the 10 sub-Saharan African countries.

## Discussion

Results of this study highlight the positive impact that expanded ART may have in sub-Saharan African countries already burdened with high numbers of AIDS orphans. We found that achieving universal ART uptake among adults may avert over 4 million maternal, paternal and double AIDS orphans over the next 10 years.

These findings underscore the critical role of ART for reducing harms associated with AIDS orphanhood in countries such as South Africa and Nigeria, where annual rates of ART uptake were projected to have the greatest impact. They also draw attention to the need for accelerated ART expansion in countries, such as Zimbabwe and Uganda, where low annual rates of ART expansion will have a comparatively reduced impact on orphanhood averted.

These results have important implications for the health and quality of life of children in sub-Saharan Africa and other HIV-endemic areas. Studies in Zimbabwe and Namibia have found that orphans experience elevated psychological distress, including symptoms of depression [3,4] Across Africa, orphans appear to have limited access to basic material goods and education, and tend to drop out of school more than non-orphans [1]. Studies in Zimbabwe have found that orphans, and particularly maternal orphans, are at elevated risk of acquiring HIV since they engage in early and unprotected sex, and have multiple sexual partners [5,6]. HIV-positive orphans have shown to have delayed access to HIV treatment and care in Uganda, reduced adherence to ART in Kenya, and poor nutritional status in Thailand [7-9]. We found that universal ART access would have a particularly positive impact on reducing the number o maternal AIDS orphans in sub-Saharan Africa. Several studies have evaluated the impact of AIDS-specific maternal mortality on orphanhood [16,21]. However, none have explored this within the context of the expansion of ART access.

Strengths and limitations of our model pertain to the Spectrum program used. Spectrum is used by UNAIDS to estimate HIV-prevalence, mortality, ART needs and orphanhood. One strength of this software is that it enables the inputting of country, age and sex-specific HIV prevalence values. In doing so, it allows modellers to consider the heterogeneity of HIV prevalence, both between and within, countries under study. However, we assumed that HIV prevalence for each country would remain constant after year 2008 due to the lack of UNAIDS data beyond that year. Since high HIV prevalence is correlated with high orphanhood, and since prevalence is declining in many sub-Saharan African countries, this assumption about a stable HIV prevalence after year 2008 may led to an overestimation of AIDS orphanhood. Use of the Estimation and Projection Package (EPP) in conjunction with Spectrum may have rectified this issue. Developers of Spectrum previously tested and validated the age and sex-specific HIV prevalence values for several countries included in our analysis (e.g. Kenya, Tanzania and Zambia) [17]. The verification of country-specific projection estimates against demographic health survey findings allowed for the generation of prevalence values that are as close as possible to actual epidemiological trends.

Program limitations relate to the detailed methodology for calculating AIDS orphans in the presence and absence of ART. For instance, there is little quantitative information regarding the effect of ART on female fertility and its effect on orphanhood. While there is an input for adult and child survival on ART, these values are fixed, and are based on a single study [19]. Another orphan modeling study assumed that women receiving ARVs had a fertility rate 50% lower than women not receiving treatment [37]. They also assumed that individuals initiating ART had a median survival 50% higher than those not on therapy. Yet, these assumptions have little empirical evidence that lend support. However, when comparing their results, the number of maternal orphans incurred in South Africa with ART intervention was similar to our findings, indicating that their methodology paralleled our own.

Discrepancies between Spectrum-based and empirical household survey estimates of orphanhood have been previously identified. Projected estimates of orphanhood have tended to be higher than empirical approximates [28,29]. This may be due either to several factors including under-reporting of deaths in household surveys, erroneously high non-AIDS related mortality rates in projection models, or the fact that foster parents sometimes claim adopted children as their natural children [28,29]. Given these reported discrepancies, it is possible that our projection model may have also over-estimated the number of orphans incurred and averted in the sub-Saharan African countries under study.

This study only indirectly considered the impact of non-adherence on HIV outcomes by means of inputting empirically obtained mortality rates. A closer examination of adherence would have been valuable given the association between adherence and mortality [38]. A systematic review of 33 cohort studies in sub-Saharan Africa found that on average one-year patient retention in ART programs was 75%, with patient attrition caused by loss to follow-up or death [39]. A more recent cohort study of 48,338 Médecins Sans Frontières patients found median patient retention to be 86% at one year [40]. These empirical studies suggest adult survival rates may be lower than what we inputted in our model, and that the projected number of orphans averted may also be slightly lower.

Another potential limitation of our analysis relates to our assumption that the TFR of women on ART would be comparable with that of the general population, while the TFR of women not on ART is depressed [41,42]. A recent study from Uganda has shown, however, that women on ART were 44% less likely to become pregnant and 70% less likely to give birth than HIV-positive women not on ART in the three years prior to the study [43]. It remains to be determined if this fertility differential remains constant over the course of the reproductive lifespan. In this case, our assumption will have slightly overestimated the TFR of women on ART, thereby overestimating the number of orphans averted through expanded access to ART. Nevertheless, as shown in the case of South Africa, even when the TFR is low, high HIV prevalence and high rates of ART use still result in a high number of maternal orphans averted. Other potential limitations in our study include our assumption that adult and child ART survival was the same for all countries may not be reflective of actual country rates.

## Conclusion

Our projection model strongly argues that expanded access to HIV treatment will have immediate preventive impact on the health and welfare of children in sub-Saharan Africa. If we are to make important gains in livelihood for future generations in Africa, expanding access to ART should be of paramount importance.

## Abbreviations

(AIDS): Acquired immune deficiency syndrome; (ART): antiretroviral therapy; (HIV): human immunodeficiency virus; (MTCT): mother-to-child transmission; (PMTCT): prevention of mother-to-child transmission; (TFR): total fertility rate; HIV/AIDS (UNAIDS): Joint United Nations Programme on HIV/AIDS; (UNICEF): United Nations Children's Fund; (USAID): United States Agency for International Development; (WHO): World Health Organization.

## Competing interests

The authors declare that they have no competing interests.

## Authors' contributions

AA conceived the study design, contributed to the demographic modeling methods, and wrote the first draft of the manuscript. CA and MJ ran the demographic projection software and contributed to the first draft of the paper. AK contributed to specialized knowledge on reproductive health issues specific countries under investigation. SK, KV, JSGM and BRSH provided critical feedback on study design and manuscript draft. All authors read and approved the final manuscript.
